# The ICR639 CPG NGS validation series: A resource to assess analytical sensitivity of cancer predisposition gene testing

**DOI:** 10.12688/wellcomeopenres.14594.1

**Published:** 2018-06-12

**Authors:** Shazia Mahamdallie, Elise Ruark, Esty Holt, Emma Poyastro-Pearson, Anthony Renwick, Ann Strydom, Sheila Seal, Nazneen Rahman

**Affiliations:** 1 Division of Genetics & Epidemiology, The Institute of Cancer Research, London, SM2 5NG, UK; 2 TGLclinical, The Institute of Cancer Research, London, SM2 5NG, UK; 3 Cancer Genetics Unit, Royal Marsden NHS Foundation Trust, London, SM2 5PT, UK

**Keywords:** next generation sequencing, cancer predisposition gene, BRCA1 and BRCA2, analytical sensitivity, genetic testing, validation, variant benchmarking

## Abstract

The analytical sensitivity of a next generation sequencing (NGS) test reflects the ability of the test to detect real sequence variation. The evaluation of analytical sensitivity relies on the availability of gold-standard, validated, benchmarking datasets. For NGS analysis the availability of suitable datasets has been limited. Most laboratories undertake small scale evaluations using in-house data, and/or rely on *in silico* generated datasets to evaluate the performance of NGS variant detection pipelines.

Cancer predisposition genes (CPGs), such as *BRCA1* and *BRCA2*, are amongst the most widely tested genes in clinical practice today. Hundreds of providers across the world are now offering CPG testing using NGS methods. Validating and comparing the analytical sensitivity of CPG tests has proved difficult, due to the absence of comprehensive, orthogonally validated, benchmarking datasets of CPG pathogenic variants.

To address this we present the ICR639 CPG NGS validation series. This dataset comprises data from 639 individuals. Each individual has sequencing data generated using the TruSight Cancer Panel (TSCP), a targeted NGS assay for the analysis of CPGs, together with orthogonally generated data showing the presence of at least one CPG pathogenic variant per individual. The set consists of 645 pathogenic variants in total. There is strong representation of the most challenging types of variants to detect, with 339 indels, including 16 complex indels and 24 with length greater than five base pairs and 74 exon copy number variations (CNVs) including 23 single exon CNVs. The series includes pathogenic variants in 31 CPGs, including 502 pathogenic variants in *BRCA1* or *BRCA2*, making this an important comprehensive validation dataset for providers of *BRCA1* and *BRCA2* NGS testing. We have deposited the TSCP FASTQ files of the ICR639 series in the European Genome-phenome Archive (EGA) under accession number EGAD00001004134.

## Introduction

For a clinical test based on next generation sequencing (NGS) to be approved for use, its performance with respect to accuracy, analytical sensitivity, analytical specificity and precision, must be evaluated ^1– 4^. Analytical sensitivity refers to the ability of a sequencing test to detect real sequence variation. The evaluation of analytical sensitivity therefore relies on the availability of gold-standard, validated, benchmarking datasets. For NGS analysis the availability of suitable datasets has been limited. Most laboratories undertake small scale evaluations, using in-house data that seldom comprehensively covers the spectra of variant types the test must detect ^5, 6^. Many laboratories also rely on *in silico* generated datasets to evaluate the performance of NGS variant detection pipelines. Whilst of value, *in silico* data cannot completely replace experimental data generated from biological samples that have been orthogonally validated ^7^.

Cancer predisposition genes (CPGs), such as *BRCA1* and *BRCA2*, are amongst the most widely tested genes in clinical practice ^6, 8– 10^. Hundreds of providers across the world are now offering CPG testing using NGS methods, either through panel, exome, or whole genome testing ^9^. Increasingly, the analysis of the data is processed separately to the generation of data and the clinical reporting of results, sometimes through outsourcing data analysis to a separate provider. This makes assessments and comparisons of analytical sensitivity even more challenging.

We have conducted CPG testing in research and clinical settings for over a decade, identifying many hundreds of pathogenic variants. We have generated extensive sequence-based data on thousands of samples using a variety of technologies including NGS methods, PCR amplification with Sanger sequencing, standard Multiplex Ligation-dependent Probe Amplification (MLPA), MLPA by NGS and Conformation Sensitive Gel Electrophoresis (CSGE). To validate the analytical sensitivity of our ISO 15189 accredited CPG NGS clinical testing pipeline, we used data from 639 individuals known to have pathogenic variants in CPGs through testing by other methods. This validation resource has proved invaluable for ensuring optimal analytical sensitivity during the initial and ongoing development of our NGS pipelines.

To assist those without access to extensive validated datasets we have put together the ICR639 CPG NGS validation series, which we present here.

The ICR639 CPG NGS validation series comprises data from 639 individuals. Each individual has sequencing data generated using the TruSight Cancer Panel (TSCP), a targeted NGS assay for the analysis of CPGs ^11^, together with orthogonally generated data showing the presence of at least one CPG pathogenic variant per individual. The set consists of 645 pathogenic variants in total. There is strong representation of the most challenging types of variants, with 339 indels, including 16 complex indels and 24 with length greater than five base pairs and 74 exon copy number variations (CNVs) including 23 single exon CNVs ( Table 1). The series includes pathogenic variants in 31 CPGs. There are 502 pathogenic variants in *BRCA1* or *BRCA2*, making this an important comprehensive validation dataset for providers of *BRCA1* and *BRCA2* NGS testing. The vast majority of variants occur in extremely high-quality sequencing data, fulfilling a Quality Sequencing Minimum (QSM) of C50_B10(85)_M20(95) ^12^. As such, it is anticipated that any accredited test provider will be able to detect these variants.

**Table 1.** Summary of pathogenic variant types by gene in the ICR639 CPG NGS validation series.

	Pathogenic Variant Type					
Gene	Base substitutions	Deletions	Insertions	Complex indels	Exon CNVs	Total
*APC*	2	1	1	0	0	4
*ATM*	3	1	1	0	1	6
*BRCA1*	85	102	24	3	30	244
*BRCA2*	86	120	30	11	11	258
*BRIP1*	4	4	3	0	0	11
*CDH1*	1	0	0	0	0	1
*CDKN2A*	2	1	0	0	0	3
*CHEK2*	0	2	0	0	4	6
*DICER1*	1	0	2	1	0	4
*EPCAM*	0	0	0	0	1	1
*FH*	2	0	0	0	1	3
*GPC3*	1	0	0	0	0	1
*HRAS*	1	0	0	0	0	1
*MEN1*	1	0	1	0	0	2
*MLH1*	4	1	2	0	1	8
*MSH2*	4	1	1	0	8	14
*MSH6*	6	2	3	0	2	13
*MUTYH*	1	0	0	0	0	1
*NF1*	1	2	0	0	1	4
*PALB2*	6	9	3	0	1	19
*PMS2*	2	1	0	0	4	7
*PTEN*	2	2	1	0	1	6
*RAD51C*	3	0	0	0	1	4
*RAD51D*	2	1	1	0	0	4
*RB1*	0	0	0	0	1	1
*RET*	3	0	0	0	0	3
*SDHB*	1	0	0	0	2	3
*SDHD*	1	0	0	0	0	1
*SMARCB1*	2	0	0	0	0	2
*TP53*	2	0	0	1	3	6
*WT1*	3	0	0	0	1	4

The dataset size and comprehensive representation of variant types that can be detected by targeted sequencing, makes the ICR639 CPG NGS validation series a valuable benchmarking resource for providers of CPG testing by NGS. The dataset may also be of value to laboratories analysing other genes, and those performing exome or genome testing which will encompass CPGs. The ICR639 CPG NGS validation series was constructed as part of the Transforming Genetic Medicine Initiative (TGMI, www.thetgmi.org) a Wellcome funded initiative that is developing frameworks and resources to facilitate genetic medicine.

## Methods

We used lymphocyte DNA from 639 individuals. The individuals were either recruited to our studies to discover and characterise disease predisposition genes, which have been approved by the London Multicentre Research Ethics Committee (05/MRE02/17, MREC/01/2/044, MREC/01/2/18), or from the TGLclinical laboratory, an ISO 15189 accredited genetic testing laboratory. Written informed consent from patients tested through TGLclinical includes use of samples for quality-control and research.

We generated high-quality targeted NGS data for the ICR639 CPG NGS validation series using the TruSight Cancer Panel (TSCP) v2 ( Supporting File 1). We prepared targeted DNA libraries from 50ng genomic DNA using the TSCP and TruSight Rapid Capture kit (Illumina, San Diego, CA, USA). We followed the manufacturer’s protocol with the exception of library enrichment pool complexity, which we performed in 48-plex. For every sample, we sequenced a final 10pM pooled library on a HiSeq 2500 platform set in Rapid-run mode following standard protocols: 96-plex pool per flow cell, HiSeq® Rapid SBS Kit v2, 101 bp paired-end dual index run, and onboard clustering using HiSeq® Rapid PE Cluster Kit v2. CASAVA v.1.8.2 was used to demultiplex and create FASTQ files per sample from the raw base call files.

To evaluate data quality, we mapped the sequencing reads to the human reference genome (GRCh37) using Stampy v.1.0.20 ^13^ with BWA v.0.7.5a ^14^ for pre-mapping. We used CoverView v.1.1.0 ^15^ to flag fragments containing the pathogenic variant, which did not fulfil a QSM ^12^ of C50_B10(85)_M20(95) for all base substitutions and indels. All samples with an exon CNV pathogenic variant passed the default settings of DECoN v.1.0.0 ^16^.

All 639 individuals also had orthogonally generated data available. These data were generated through either PCR amplification with Sanger sequencing ^17^, standard MLPA or MLPA by NGS ^11, 18^.

Annotation of base substitutions and indels follows Clinical Sequencing Notation (CSN) v.1.0 ^19^ using the RefSeq mRNA transcripts. For all genes except *WT1* the coding annotation (c.) starts at 1, the A of the ATG translation initiation codon. For *WT1*, c.1 is the A of the first in-frame AUG translation initiation codon and the KTS exon 9 sequence is included. We used Ensembl ENST transcripts from release 65 for exon CNV annotation as RefSeq mRNA transcripts do not specify intron/exon boundaries. All exon CNVs are described using the following notation “Exon X deletion/duplication” for single exon CNVs and “Exon X-Y deletion/duplication” for exon CNVs involving more than one exon, where X specifies the number of the first exon involved in the exon CNV with respect to the transcript, Y specifies the number of the last exon involved in the exon CNV with respect to the transcript, and deletion or duplication is specified as appropriate. For all genes except *BRCA1* exon numbering is consecutive from the first non-coding exon in the transcript. For *BRCA1* we use the conventional clinical numbering system that does not include exon 4.

We provide the left-aligned CHR, POS, REF and ALT information according to GRCh37 for base substitutions and indels to allow comparison with Variant Calling Format (VCF) files. All exon CNVs were validated by MLPA. We provide the most 5’ and most 3’ genomic coordinates of the exons involved in the exon CNV according to the exon numbering of the specified transcript. Of note, these are not the actual breakpoints; standardly neither MLPA nor targeted NGS data provides breakpoint sequence information for exon CNVs.

## Dataset

The ICR639 CPG NGS validation series includes data from 639 individuals. Six individuals have two different pathogenic variants, so the dataset contains a total of 645 pathogenic CPG variants ( Supporting File 2). The pathogenic variants occur in 31 different genes that are all proven disease-causing genes and are routinely tested in clinical practice ^6, 8– 10^. The series includes 502 pathogenic variants in *BRCA1* or *BRCA2*, which are the most commonly tested CPGs, and 43 pathogenic variants in the Lynch syndrome genes ( *MLH1*, *MSH2*, *MSH6*, *PMS2* and *EPCAM*).

All 645 pathogenic CPG variants are different and together they cover the variant types routinely detected and reported in clinical genetic testing ( Table 1). There are 232 base substitutions, 323 insertions or deletions, 16 complex indels and 74 exon CNVs. Of note, the set include 24 insertions or deletions with length greater than five base pairs and 23 single exon CNVs, two challenging variant classes to detect in NGS data.

The ICR639 CPG NGS validation series comprises high quality sequencing data. For 561 of the 571 base substitutions and indels (98%), the fragment containing the variant fulfilled a QSM of C50_B10(85)_M20(95) ^12^. This represents a minimum quality requirement whereby 100% of bases in the fragment had at least 50x depth of coverage with a base quality score of ≥10 in at least 85% of reads and a mapping quality score of ≥20 in at least 95% of reads. For the remaining ten pathogenic variants, the fragment containing the variant did not meet the QSM requirement for either the base quality (n=5) or the mapping quality (n=5). All fragments fulfilled the coverage requirement. We include these variants to allow evaluation of variant detection performance in data with suboptimal base or mapping quality, as such data is commonly encountered in genetic testing. The sequencing data for all 74 exon CNVs fulfilled the minimum quality requirements of DECoN, a batch-based exon CNV calling tool ^16^, namely a minimum correlation of 0.98 with other samples in its batch and a minimum median coverage metric of 100 across all exons in the target. The ICR639 CPG NGS validation series is thus a high-quality sequencing dataset and users are expected to detect all pathogenic variants in CPG(s) of relevance to their pipeline.

We have previously made freely available other datasets that groups may find useful in conjunction with the ICR639 CPG NGS validation series. For example, we generated TSCP data for the NIST-led Genome in a Bottle (GIAB) Consortium reference material (RM) 8398 ^15, 20^. We have also made available the ICR142 exome validation series ^17^ and ICR96 exon CNV validation series ^11^. These resources allow evaluation of both sensitivity and specificity, for small variants and exon CNVs respectively. Of note, 50 exon CNVs are included in both the ICR96 exon CNV validation series and the ICR639 CPG NGS validation series.

## Data availability

We have deposited the TSCP FASTQ files for all 639 individuals in the European Genome-phenome archive (EGA). The accession number is EGAS00001002993. Details of how to access the data is available at EGA or from www.icr.ac.uk/icr639.

The individual level genetic data on EGA is under managed access in line with general recommendations for use of patient information, the specific consent obtained for use of data from these samples and our institutional data access committee. The ICR-GSR data access application form should be completed and returned to rahmanlab@icr.ac.uk. Applications will only be accepted electronically. Access to the data will require the completion of a Data Access Agreement. Any queries regarding access procedures or completion of the forms should be sent to rahmanlab@icr.ac.uk.

Supporting data files have been archived on Open Science Framework: http://doi.org/10.17605/OSF.IO/N2VWR ^21^ under a CC0 1.0 Universal licence.

- **Supporting File 1. TSCP targeted BED file.** Targets of the Illumina TruSight Cancer Panel (TSCP) v2 in BED file format.
- **Supporting File 2. Pathogenic variants in the ICR639 CPG NGS validation series.** The ICR639 CPG NGS validation series: a resource to assess analytical sensitivity of cancer predisposition gene testing

The description of the column headings are given below:

- **SampleID** – sample ID in the ICR639 CPG NGS validation series
- **AnnotationTranscript** – the transcript used to annotate the variant, either the RefSeq NM ID or the Ensembl v65 ENST ID
- **Gene** – HGNC symbol
- **ReportedVariant** – Base substitutions and indels are in accordance with CSN v.1.0. Exon CNVs are described with notation “Exon X deletion/duplication” for single exon CNVs and “Exon X-Y deletion/duplication” for multi-exon CNVs, where X is the first exon and Y the last exon involved and deletion/duplication as appropriate
- **VariantType** – “bs”, “del”, “ins”, “complex”, or “exonCNV” for base substitutions, deletions, insertions, complex indels, or exon CNV variants, respectively
- **Zygosity** – “heterozygous” a pathogenic variant that is present on only one allele
- **AdjacentVariant** – annotation according to CSN v.1.0 if a variant was detected adjacent to the reported variant, “.” if no adjacent variant was detected
- **QSMFragmentResult** – “PASS” if the fragment containing the variant fulfilled QSM C50_B10(85)_M20(95), “FLAG – BaseQuality” if at least one position in the fragment containing the variant did not fulfil B10(85), “FLAG – MappingQuality” if at least one position in the fragment containing the variant did not fulfil M20(95), “.” if the variant was an exon CNV
- **CHR** – chromosome
- **POS** – the left-aligned position in GRCh37 coordinates, “.” if the variant was an exon CNV
- **REF** – the reference allele in GRCh37, “.” if the variant was an exon CNV
- **ALT** – the alternative allele, “.” if the variant was an exon CNV
- **5PrimeExon37** – most 5’ genomic coordinate of most 5’ exon in GRCh37
- **3PrimeExon37** – most 3’ genomic coordinate of most 3’ exon in GRCh37

Researchers and authors that use the ICR639 CPG NGS validation series should reference this paper and should include the following acknowledgement: "This study makes use of the ICR639 CPG NGS validation series data generated by Professor Nazneen Rahman’s team at The Institute of Cancer Research, London as part of the TGMI”.
