# Supplementary material for: The ICR639 CPG NGS validation series: A resource to assess analytical sensitivity of cancer predisposition gene testing
Source: Wellcome Open Res. 2018 Jun 12;3:68. [Version 1] doi: 10.12688/wellcomeopenres.14594.1 (PMC6081973; doi:10.12688/wellcomeopenres.14594.1)
Supplement: Peer review history

# Peer review: Reviewer response for version 1

**DOI:** 10.21956/wellcomeopenres.15889.r33501

**Published:** 2018-08-03

**Authors:** Rosamaria Pinto

**Affiliations:** 1 Pharmacogenetics and Molecular Diagnostic Unit, IRCCS Istituto Tumori "Giovanni Paolo II", Bari, Italy

Rahman *et al*., in this study, analyzed cancer predisposition genes (CPGs) using NGS methods in 639 individuals and offering a new important dataset of CPG pathogenic variants for diagnostic community.

 The paper is well written and clear in its meaning and analysis. In my opinion the article can be accepted for indexing.

I confirm that I have read this submission and believe that I have an appropriate level of expertise to confirm that it is of an acceptable scientific standard.

# Peer review: Reviewer response for version 1

**DOI:** 10.21956/wellcomeopenres.15889.r33357

**Published:** 2018-07-02

**Authors:** Conxi Lázaro, Lídia Feliubadaló

**Affiliations:** 1 Hereditary Cancer Program, Catalan Institute of Oncology (ICO), Bellvitge Institute for Biomedical Research (IDIBELL), Barcelona, Spain; 2 Hereditary Cancer Program, Catalan Institute of Oncology (ICO), Bellvitge Institute for Biomedical Research (IDIBELL), Barcelona, Spain

The manuscript is clearly written overall and the provided dataset will be a valuable tool for the diagnostics community, specially for those working on hereditary cancer. As the biological samples are not offered, the wetlab part of the diagnostic validation process of any reader’s diagnostics service will need own samples. However, the data analysis step has accquired paramount importance in next generation sequencing techniques. Thus, increasing the dataset size for the validation of the NGS data analysis step allows for a better assessment for the sensitivity and specificity of the technique.

 Information regarding the methods used to obtain the sequence data and its quality is adequate and the mutation list is detailed. Nevertheless, I have missed some information on the results of the analytical sensitivity assessment of the authors’ lab, and the criteria used to choose the dataset samples and variants.

**Minor points:**

***P5.***The dataset is described as a collection of FASTQs of sequence data from patients and genes where 639 pathogenic variants have been detected by orthogonal techniques, but I cannot find a paragraph explaining if all or some of these mutations have been detected with the described NGS pipeline too. This information would be also very useful. If any of the mutations were not found, a short explanation of the suspected reason for the false negative would be also welcome.

***P5.***The dataset includes variants in regions with suboptimal base quality or mapping quality. Have the authors in their research and clinical experience with previous techniques detected some point mutation located in the region of interest of the TruSight Cancer Panel, that have not been able to detect with the Panel? If any, are they in the dataset or left aside?

We confirm that we have read this submission and believe that we have an appropriate level of expertise to confirm that it is of an acceptable scientific standard.
